# An Old Fashioned Medical Meeting

**Published:** 1916-01

**Authors:** 


### An Old Fashioned Medical Meeting.

A while ago, such a meeting was held in a large city. It was a home talent evening, with no peasantry growing impatient for the arrival of the recently fed aristocracy and the distinguished speaker from distant parts. There were no statistics, not much pathology, only allusions to surgery, little that could be called original, drugs were mentioned as producing certain results, without scorn or apology for therapeutic faith. Various practical items of observed phenomena and carefully conceived methods of treatment were contributed and, in the free discussion, more facts were elicited and memories refreshed even when nothing brilliantly new was offered. Men left the meeting stimulated to further observation and study and with hints of much wider applicability than the special subject of the evening's program. We do not wish to be understood as condemning the modern scientific paper, still less as opposing the instillation of fresh ideas from without nor the proper reception of a guest. But, once in a while, let us hark back to the customs of our fathers and have a plain, old fashioned practical medical meeting.

---

As has been previously stated, the BUFFALO MEDICAL JOURNAL is regarded as, practically, a co-operative professional institution. In gauging the size of the Journal and other factors involving expense, as liberal an estimate is made as is practicable, with reference to income. We would, therefore remind subscribers in arrears that a prompt payment of dues means a better journal. This is true not only in the obvious, business sense, but because the attention to purely business matters necessarily detracts from the amount of time and energy available for the proper conduct of the journal.

---

THE MEDICAL ASSOCIATION OF CENTRAL NEW YORK has resumed publication of its proceedings, after an interim of several years. So far as possible, sample copies of the present issue are mailed to members not already subscribers to the BUFFALO MEDICAL JOURNAL. Subscriptions should be sent in accordance with the standing notice at the beginning of the editorial department. Dr. Chas. G. Stockton's article on the Treatment of Cardio-vascular Renal Disease was contributed independently of action on the part of the Association and was published in the December issue. Subscriptions may date from that issue, if desired, and will be filled in order, up to the limit of copies available. The stenographic minutes of the meeting will be printed within the next few issues. Certain of the original matter on the program is reported as not available for publication, either because papers were not prepared at all or because the material was presented in a form that could not be reduced to writing. The Journal can guarantee only that matter will be published as promptly as possible subject to submittance by authors. While the Journal welcomes this opportunity to extend its usefulness as a professional medium for Western New York, it does not in any sense seek it to further its own welfare, even indirectly. Thus, it should be emphasized that subscription is absolutely voluntary and that no further solicitation of subscriptions will be made.

